# Keratin 17 Promotes T Cell Response in Allergic Contact Dermatitis by Upregulating C–C Motif Chemokine Ligand 20

**DOI:** 10.3389/fimmu.2022.764793

**Published:** 2022-02-01

**Authors:** Yixin Luo, Zhenlai Zhu, Bing Li, Xiaocui Bai, Hui Fang, Pei Qiao, Jiaoling Chen, Chen Zhang, Dalong Zhi, Erle Dang, Gang Wang

**Affiliations:** Department of Dermatology, Xijing Hospital, Fourth Military Medical University, Xi’an, China

**Keywords:** allergic contact dermatitis, contact hypersensitivity, keratinocytes, keratin 17, CCL20, T cell infiltration

## Abstract

Allergic contact dermatitis (ACD) is a delayed-type hypersensitivity response to skin contact allergens in which keratinocytes are critical in the initiation of early responses. Keratin 17 (K17) is a cytoskeletal protein inducible under stressful conditions and regulates multiple cellular processes, especially in skin inflammatory diseases; however, knowledge regarding its contribution to ACD pathogenesis remains ill defined. In the present study, we clarified the proinflammatory role of K17 in an oxazolone (OXA)-induced contact hypersensitivity (CHS) murine model and identified the underlying molecular mechanisms. Our results showed that K17 was highly expressed in the lesional skin of ACD patients and OXA-induced CHS mice. Mice lacking K17 exhibited alleviated OXA-induced skin inflammation, including milder ear swelling, a reduced frequency of T cell infiltration, and decreased inflammatory cytokine levels. *In vitro*, K17 stimulated and activated human keratinocytes to produce plenty of proinflammatory mediators, especially the chemokine CCL20, and promoted keratinocyte-mediated T cell trafficking. The neutralization of CCL20 with a CCL20-neutralizing monoclonal antibody significantly alleviated OXA-induced skin inflammation *in vivo*. Moreover, K17 could translocate into the nucleus of activated keratinocytes through a process dependent on the nuclear-localization signal (NLS) and nuclear-export signal (NES) sequences, thus facilitating the activation and nuclear translocation of signal transducer and activator of transcription 3 (STAT3), further promoting the production of CCL20 and T cell trafficking to the lesional skin. Taken together, these results highlight the novel roles of K17 in driving allergen-induced skin inflammation and suggest targeting K17 as a potential strategy for ACD.

## Introduction

Allergic contact dermatitis (ACD) is a common T cell-mediated inflammatory skin disease that is induced by topical exposure to haptens or antigens, which activate antigen-specific T cells and result in intensely itching erythema and eczema (1, 2). The pathophysiology of ACD is complex and involves two phases: the sensitization phase and the elicitation phase (3). In the elicitation phase, antigen-specific T cells are recruited to inflammatory sites and activated by antigens (4). Keratinocytes are among the pivotal factors responsible for inflammation and are also activated upon re-exposure to antigen and hapten sensitization (5). Activated keratinocytes produce various keratinocyte-derived proinflammatory chemokines, such as C-X-C motif chemokine ligand 1 (CXCL1), C-C motif chemokine ligand 17 (CCL17), CCL27, and CCL20, which activate endothelial cells and recruit multiple antigen-specific T cells to participate in the delayed response (6, 7). Moreover, previous studies have demonstrated that CD4^+^ and CD8^+^ T cells are important mediators of the immune response to contact allergens (8–10). However, although the cross-talk between chemokines secreted by keratinocytes and T cell chemotaxis reportedly plays a pivotal role in the development of chronic inflammatory skin diseases, the detailed mechanism has not been well elucidated.

Keratin17 (K17) is a member of the keratin family, which is abnormally expressed in epidermal keratinocytes in multiple inflammatory skin diseases (11, 12). Studies published in recent decades have implied roles of the multifunctional protein K17 in many cellular processes, including cell growth and proliferation and skin inflammation (13, 14). As shown in our previous studies, elevated K17 expression in psoriatic epidermal keratinocytes upregulates the expression of multiple proinflammatory cytokines and chemokines and forms a K17-T cell-cytokine autoimmune loop that participates in the development of psoriasis (15–18). Additionally, recent studies found that the cytoskeletal protein K17 localizes in the nucleus of tumor cells and participates in the development of tumors, which are traditionally thought to be located and function exclusively in the cytoplasm (19, 20). This new biological phenomenon of intermediate filaments raises the possibility that keratin regulates additional cellular processes in various diseases (21). However, the pathogenic contribution of K17 to the development of ACD remains unclear.

In this study, we investigated the proinflammatory role and underlying mechanisms of K17 in OXA-induced ACD-like skin inflammation using *K17*-knockout (KO) mice. Our data showed that ACD-like skin inflammation was significantly attenuated in K17-deficient mice compared with wild-type (WT) mice. Moreover, K17 enhanced T cell migration by upregulating CCL20 expression in keratinocytes in a process dependent on K17 translocation and interaction with signal transducer and activator of transcription 3 (STAT3) and mediated by the NLS and NES sequences of K17, which then drives T cell infiltration and contributes to ACD development. These results provide novel insight into the potential role of K17 in ACD pathogenesis.

## Materials and Methods

### Collection of Specimens From Patients and Controls

All skin biopsies were obtained at the Department of Dermatology, Xijing Hospital (Shaanxi, China). All procedures were approved by the Declaration of Helsinki Principles of the Fourth Military Medical University. All subjects signed an informed consent form, and full-thickness skin specimens were collected from the lesional skin of ACD patients.

### Mice

Female mice (C57BL/6J; 8-10 weeks old) used in the experiments were purchased from the Department of Laboratory Animal Medicine of the Fourth Military Medical University. K17 knockout (K17 KO) mice were kindly provided by Prof. Pierre A. Coulombe (Johns Hopkins University, Baltimore, MD 21205, USA). All animal experiments were performed in accordance with the guidelines of the Fourth Military Medical University.

### Animal Experiments

C57BL/6J and K17 KO mice were sensitized with 20 µL of 3% OXA (Sigma-Aldrich, St. Louis, MO, USA) dissolved in anhydrous ethanol (EtOH) on the ears. After 5 days, the ears were repeatedly challenged with 10 µL of 1% OXA on one ear, and the opposite ear was treated with EtOH. For neutralization of the CCL20 experiment, C57BL/6J mice were intraperitoneally (i.p.) injected with CCL20-neutralizing monoclonal antibody (CCL20 mAb, 100 µg/mouse, R&D Systems, USA), or rat IgG isotype control antibody (100 µg/mouse, R&D Systems, USA). Ear thickness was measured by a micrometer caliper at the indicated time points (δear thickness & δdermal thickness = right-ear thickness – left-ear thickness).

### Cell Culture and Transfection

Human HaCaT KC cells (HaCaT cells; American Type Culture Collection, Manassas, VA, USA) were cultured in Dulbecco’s modified Eagle medium (Gibco, Grand Island, NY, USA) supplemented with 10% fetal bovine serum (Invitrogen, Carlsbad, CA, USA) and maintained in a 5% CO_2_ humidified atmosphere at 37°C. Cells were stimulated with IL-17, IL-22, TNF-α, and interferon (IFN)-γ (50, 20, 50, and 20 ng/mL, respectively) (PeproTech, Rocky Hill, NJ, USA) in 6-well plates for 48 h, with phosphate-buffered saline (PBS) used as a negative control. The nuclear-transport inhibitor leptomycin B (LMB, S1726, Beyotime Biotechnology, Shanghai, China) (20 nmol/mL) was added 3 h before cells were harvested to block the nuclear transport of K17. Additionally, HaCaT cells were transfected with pEGFP-N1-K17, pEGFP-N1-K17-NLS^−/−^, pEFP-N1-K17-NES^−/−^ plasmids, or short-interfering RNA (siRNA) targeting *K17* and *STAT3* using Lipofectamine 3000 (Invitrogen). siRNA sequences for *K17* and *STAT3* are listed in **Supplementary Table S1** online.

### Cell Culture of Human Primary Keratinocytes

Human primary keratinocytes were obtained from three patients (aged from 8 to 30 years) foreskins after urological surgery from department of urology, Xijing Hospital. Human primary keratinocytes were maintained in keratinocytes complete media (KCM, ThermoFisher): EpiLife medium +60µM calcium/EpiLife Defined Growth Supplement (EDGS, ThermoFisher) and cells were cultured with 50U/ml penicillin-streptomycin. Keratinocytes were transfected with STAT3 siRNA, K17 siRNA and K17 overexpressing plasmid in 6-well plates at passages from 2 to 4.

### Real-Time Quantitative Reverse Transcription Polymerase Chain Reaction (qRT-PCR)

TRIzol Reagent (Invitrogen) was used to extract total RNA from cells or mouse tissues, and total RNA (2 µg) was subjected to reverse transcription into cDNA using the PrimeScript RT-PCR kit (Takara, Seoul, Korea). Real-time PCR was performed using SYBR Premix Ex Taq II (Takara) with a CFX384 detection system (Bio-Rad Laboratories, Hercules, CA, USA). Primer sequences are given in **Supplementary Table S2** online, and relative gene expression levels were calculated using the comparative Ct (2 ^−ΔΔCT^) method.

### Western Blot

Whole proteins in HaCaT cells were extracted in radioimmunoprecipitation assay buffer (Runde Biologicals Ltd., Yichun, China) supplemented with 1 mM phenylmethylsulfonyl fluoride. Nuclear and cytoplasmic proteins were separated using a nuclear and cytoplasmic protein extraction kit (#P0027; Beyotime Biotechnology, Shanghai, China). Proteins were loaded onto 10% sodium dodecyl sulfate polyacrylamide gels and blotted onto polyvinylidene fluoride membranes, which were blocked with 5% bovine serum albumin for 1 h and then incubated with the following corresponding primary and horseradish peroxidase (HRP)-conjugated secondary antibodies: anti-K17 (1:1000; ab53707; Abcam, Cambridge, UK), anti-lamin A/C (1:1000; Proteintech, Phoenixville, PA, USA), anti-CCL20 (1:100; ab9828; Abcam), anti-STAT3 (1:1000; #124H6; Cell Signaling Technology) and anti-β-actin (1:5000; CoWin Biosciences, Cambridge, MA, USA). Specific bands were detected using an enhanced chemiluminescence substrate (Thermo Fisher Scientific, Waltham, MA, USA).

### Skin Histopathology

OXA-induced mouse tissue samples were fixed with 4% paraformaldehyde and embedded in paraffin, followed by staining of the 8-µm paraffin sections with hematoxylin and eosin (H&E; Wako, Osaka, Japan). Slides were scanned using a slide scanner (HAMAMATSU Photonics, Iwata City, Japan) and analyzed using NDP2 viewer software (HAMAMATSU Photonics).

### Preparation of a Single-Cell Suspension and Flow Cytometry

Mouse ear specimens were mechanically excised, cut into several slices, digested with collagenase type IV (2 mg/mL; Worthington Biochemical Corp., Lakewood, NJ, USA) in Roswell Park Memorial Institute (RPMI)-1640, 1 mg/mL DNase I (Sigma-Aldrich), and 2% fetal calf serum (HyClone, San Angelo, TX, USA) for 40 min at 37°C. Cells in RPMI medium were then strained with a 70-µm filter, and the single-cell suspension was collected and stained for flow cytometric analysis. Human CD4^+^ and CD8^+^ T cells were separated from peripheral blood mononuclear cells (PBMCs) using flow cytometry.

The following fluorophore-labeled monoclonal antibodies were used. CD4 (clone GK1.5) and CD8 (clone 53-6.7) antibodies were used to sort CD4^+^ and CD8^+^ T cells, respectively. 7-Aminoactinomycin D (7-AAD; BioLegend, San Diego, CA, USA) was used to stain dead cells before analysis, and CountBright absolute counting beads (Invitrogen) were used to calculate the number of recovered cells. Flow cytometry was performed using the FACSCalibur system (BD Biosciences, Franklin Lakes, NJ, USA), and data were analyzed with FlowJo software (FlowJo v10; TreeStar, Ashland, OR, USA).

### Enzyme-Linked Immunosorbent Assay (ELISA)

CCL20 levels were measured using an ELISA kit (Elabscience, Houston, TX, USA) according to manufacturer’s instructions.

### Chemotaxis Assay

Transwell chambers (8-µm pore size, 24 wells; Millicell; Millipore, Billerica, MA, USA) were used to perform the chemotaxis assays. For this experiment, 1 × 10^5^ CD4^+^ and CD8^+^ T cells separated from PBMCs from healthy donors were separately seeded in the upper chamber, and 650 μL of HaCaT cell suspensions from different treatments were cocultured in the bottom chamber. After 4 h incubation, cells in the lower Transwell membrane were stained and calculated to determine the effects of CCL20 on CD4^+^ and CD8^+^ T cell trafficking by using a Countess II hemocytometer (Thermo Fisher Scientific, Waltham, MA, USA). All experiments were independently performed in triplicate.

### Immunofluorescence and Immunohistochemical (IHC) Staining

Lesional skin biopsy specimens from AD patients or OXA-induced mouse tissue specimens were fixed with a 12% formaldehyde solution and embedded in paraffin. For immunofluorescence staining, cells or skin biopsy specimens were incubated with the corresponding primary antibodies at 4°C overnight as follows: anti-K17 (1:1000; ab53707; Abcam), anti-CCL20 (1:300; ab9829; Abcam), anti-CD3 (1:500; ab16669; Abcam), anti-CD4 (1:500; ab183685; Abcam), anti-CD8 (1:500; ab22378; Abcam), anti-STAT3 (1:500; #124H6; Cell Signaling Technology) and anti-p-STAT3 (1:100; #9145; Cell Signaling Technology). After three washes with PBS, Cy3- or fluorescein isothiocyanate-conjugated secondary antibodies (1:200; BioLegend) were added, and Hoechst 33258 (Solarbio Technology, Beijing, China) was applied to label the nuclei. Samples were detected by a confocal microscope (LSM880; Carl Zeiss). For IHC staining, samples were incubated with 0.3% H_2_O_2_ for 10 min prior to staining with the corresponding primary antibodies: anti-K17 (1:1000; ab53707; Abcam) and anti-CCL20 (1:300; ab9829; Abcam) at 4°C overnight. Sections were subsequently incubated with an HRP-labeled goat anti-mouse/rabbit antibody (CoWin Biosciences) for 1 h at room temperature. 3,3’-Diaminobenzidine (Gene Tech, Shanghai, China) was used to detect biotinylated antibodies.

### Time-Lapse Live-Cell Imaging

HaCaT cells were seeded in 15-mm glass-bottom dishes precoated with a poly-L-lysine solution (P4832; Sigma-Aldrich) and incubated for 24 h until 80% to 90% confluence. Cells were transfected with pEGFP-N1-K17, pEGFP-N1-K17-NLS^−/−^ or pEFP-N1-K17-NES^−/−^ plasmids for 24 h and washed with PBS to remove the suspended cells. Fresh PBS was then added, and K17 translocation was observed using a confocal microscope (LSM880; Carl Zeiss, Oberkochen, Germany) at 37°C and under a 5% CO_2_ humidified atmosphere.

### Co-Immunoprecipitation (Co-IP)

Cells were collected after specific treatments, and Co**-**IP experiments using anti-K17 (sc-393002; Santa Cruz Biotechnology, Dallas, TX, USA), anti-STAT3 (#9139; Santa Cruz Biotechnology), and anti-IgG (Beyotime Biotechnology) were performed with Protein A/G PLUS-Agarose (sc-2003C; Santa Cruz Biotechnology) according to the manufacturer’s instructions. Whole-cell lysates were purified in lysis buffer and incubated with anti-K17 or anti-STAT3 on a rocker platform for 3 h, followed by incubation with Protein A/G PLUS-Agarose at 4°C overnight. Pellets were washed four times with PBS, the supernatant was discarded, and the pellets were resuspended with 1× electrophoresis sample buffer, followed by immunoblot analysis.

### Statistical Analyses

Each experiment was performed at least in triplicate. GraphPad Prism software (v.7.0; GraphPad Software, La Jolla, CA, USA) was used for statistical analyses. Statistical significance was determined using Student’s un-paired two-tailed t test or one-way analysis of variance as indicated in the legend (*P < 0.05, **P < 0.01, ***P < 0.001, ****P < 0.0001). FlowJo v10 was used for flow cytometry data analyses. The number of sampled units, n, is indicated in the figure legends.

## Results

### K17 Expression Is Elevated in the Lesional Skin of ACD Patients and OXA-Induced CHS Mice

**Figure 1:**
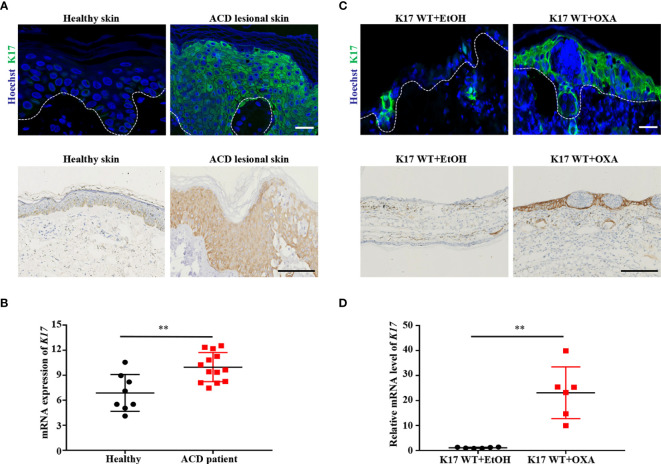
K17 expression is increased in the lesional skin of ACD patients and OXA-induced mice. **(A)** Immunofluorescence and IHC staining of K17 in the lesional skin of ACD patients (*n* = 5) and healthy controls (*n* = 5). K17 (green) and Hoechst (blue). Scale bar, 10 μm (upper panel) and 100 μm (lower panel). **(B)** The gene expression profile in the GEO dataset (GDS2935) was analyzed for the expression of K17 in both ACD patients (*n* = 13) and healthy controls (*n* = 8). **(C)** Immunofluorescence and IHC staining of K17 in ear sections from OXA-induced mice, with ethanol used as a control. *n* = 5 per group. K17 (green) and Hoechst (blue). Scale bar, 10 μm (upper panel) and 100 μm (lower panel). **(D)** Relative mRNA expression of *K17* in mouse ear skin specimens was analyzed by qRT-PCR at 24 h after OXA challenge. Data represent the mean ± SD (*n* = 6) **p < 0.001. All the bars represent at least average of three independent experiments.

K17 is involved in numerous inflammatory skin diseases and plays a particularly prominent role in immune regulation. However, few studies have examined the function of K17 in the development of ACD. We first validated the expression of K17 in lesional skin samples obtained from ACD patients and normal donors. Immunofluorescence and IHC staining results showed that K17 was highly expressed in the epidermis of ACD patients relative to normal controls (**Figure 1A**). Furthermore, GEO datasets analysis confirmed that the K17 mRNA level was higher in ACD patient lesional skin (*n* = 13) than those in healthy controls (*n* = 8) (GDS2935) (**Figure 1B**). Thereafter, we assessed K17 expression in an OXA-induced murine ACD-like model, with EtOH used as a control. Consistent with the results in ACD patients, the protein and mRNA levels of K17 were both significantly elevated in OXA-induced CHS mice relative to controls (**Figures 1C, D**). These results demonstrated that K17 was overexpressed in the epidermis of both ACD patients and OXA-induced CHS mice, prompting us to speculate that K17 might be involved in ACD pathogenesis.

### K17 Deficiency Attenuates OXA-Induced CHS Response

**Figure 2:**
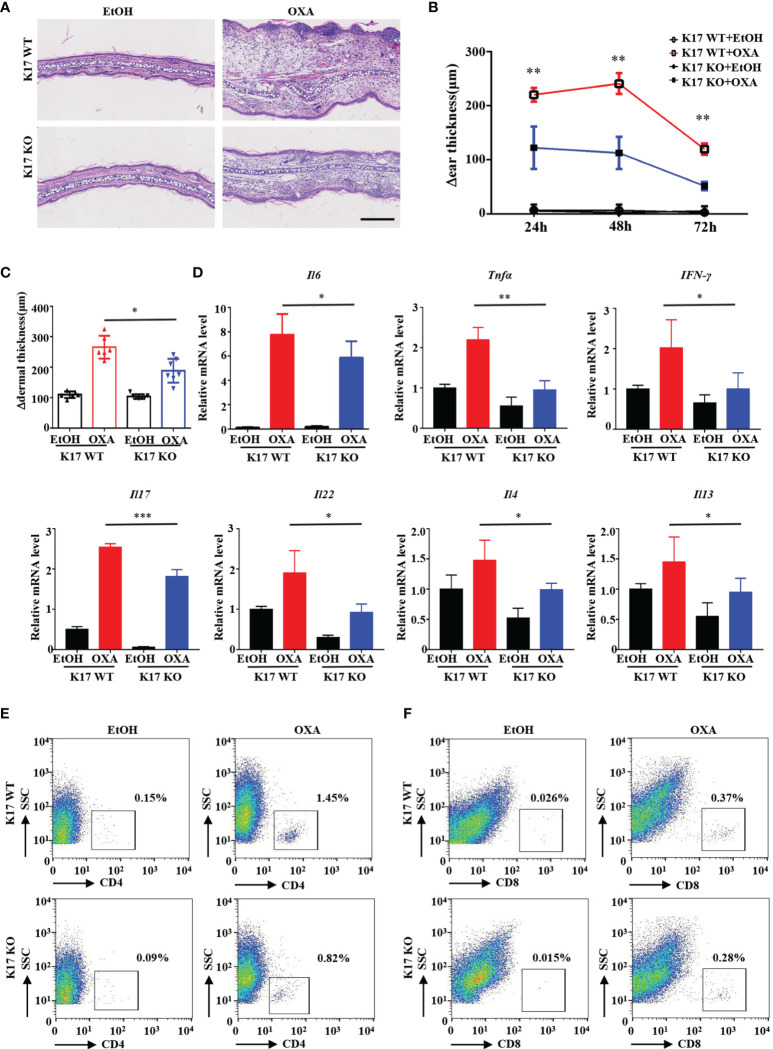
K17 deficiency attenuates OXA-induced ACD-like skin inflammation. **(A)** H&E staining of sections from K17 KO and K17 WT mice at 24 h after challenge with OXA or ethanol. Scale bar, 10 μm. The study involved 3-5 mice per group and 3 independent experiments. Examination of **(B)** Δear thickness and **(C)** Δdermal thickness, *n* = 3 per group **(D)** Relative *Il6*, *Tnfa*, *IFN-γ*, *Il17*, *Il22*, *Il4* and *Il13* mRNA expression in the mouse epidermis, *n* = 5 per group. The percentage of **(E)** CD4^+^ T cells and **(F)** CD8^+^ T cells gated from live cells was quantified using flow cytometry. Data represent the mean ± SD (*n* = 3). *p < 0.05, **p < 0.01, ***p < 0.01. All the bars represent at least average of three independent experiments.

To elucidate whether K17 is required for ACD development, we used K17 KO mice to establish the CHS murine model through topical application of OXA. Immunofluorescence and IHC staining revealed little expression of K17 in K17 KO mice, even after OXA challenge (**Supplemental Figures S1A, B**). We then compared the effects of K17 in K17 KO and WT mice treated with OXA. H&E staining showed that fewer inflammatory cells and a thinner appearance of the ears in K17 KO versus WT mice upon challenged with OXA (**Figure 2A**). Additionally, the Δear thickness and Δdermal thickness of K17 KO mice were ~50% thinner than those observed in WT controls (**Figures 2B, C**). Moreover, mRNA expression of the proinflammatory cytokines *Il6*, *Tnfa*, *IFN-γ*, *Il17*, *Il22*, *Il4* and *Il13* were significantly decreased in K17 KO CHS mice (**Figure 2D**). Previous studies have found that CD4^+^ and CD8^+^ T cells are abundant in the lesional skin of ACD patients (22). We then separated single-cell suspensions from mouse skin specimens (**Supplemental Figure S2A**) and determined T cell infiltration using fluorescence-activated cell sorting. As shown in **Figures 2E, F** and **Supplemental Figures S2B, C**, we detected fewer CD4^+^ and CD8^+^ T cells in K17 KO mice than in WT mice at 24 h post elicitation. Consistently, immunofluorescence results also showed a reduced frequency of CD4^+^, CD8^+^, and CD3^+^ T cell infiltration in K17 KO mice upon treatment with OXA compared with WT mice (**Supplemental Figures S2D–F**). These findings confirmed that the inflammatory response and T cell infiltration were significantly suppressed in K17 KO mice, indicating an essential role of K17 in the OXA-induced CHS response.

### K17 Induces Keratinocyte-Mediated Chemotaxis of T Cells by Upregulating CCL20 Expression in Keratinocytes

**Figure 3:**
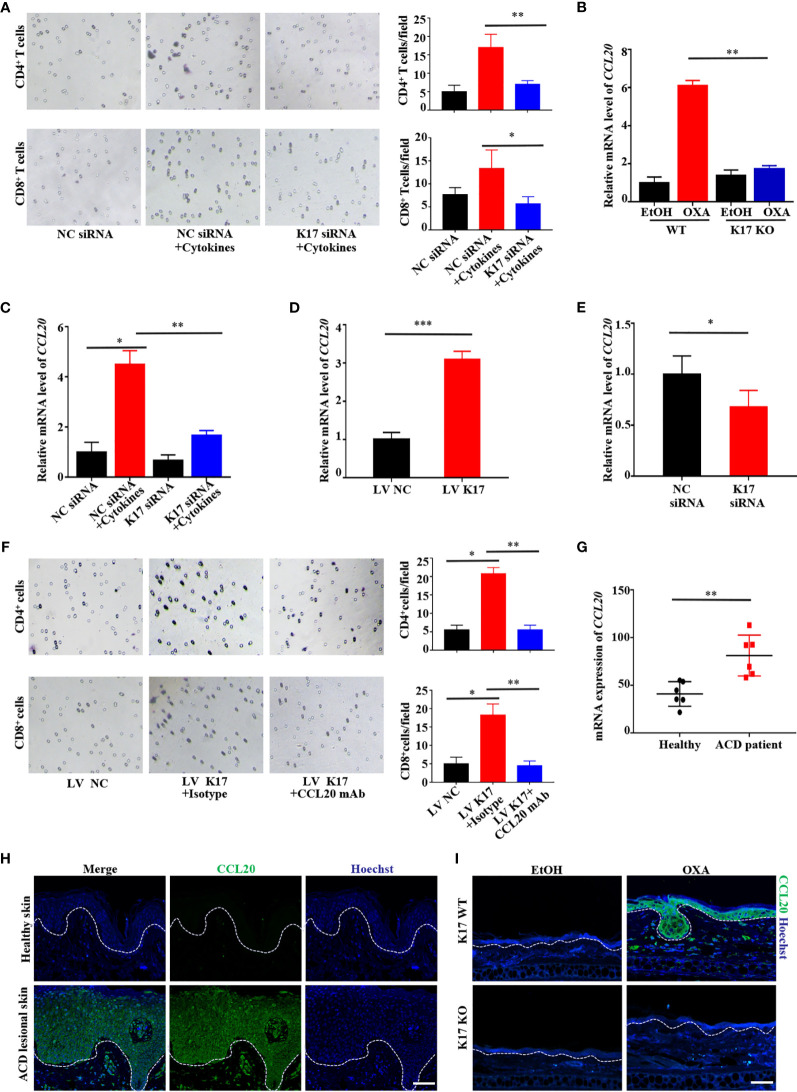
K17 induces keratinocyte-mediated T cell chemotaxis by upregulating CCL20 expression in keratinocytes. **(A)** The chemotaxis effect of K17 was determined by performing crystal violet staining of the lower Transwell membrane in cytokine- and *K17*-siRNA-treated HaCaT cells. **(B)** Relative mRNA expression of *CCL20* in K17 KO and WT mice treated with OXA, n = 3 (mean ± SD). **(C)** Relative mRNA expression of CCL20 in cytokines- and K17-siRNA-treated HaCaT cells. **(D, E)** Relative *CCL20* expression in HaCaT cells treated with pCMV6-XL5-K17 (LV K17) or *K17* siRNA was analyzed by qRT-PCR. **(F)** The chemotaxis effect of K17 was determined by performing crystal violet staining of the lower Transwell membrane in K17-overexpressing cells. **(G)** Gene-expression profile in the GEO dataset (GSE60028) was analyzed for the mRNA expression of *CCL20* in ACD patients (*n* = 6) and healthy controls (*n* = 6). **(H)** Immunofluorescence staining for CCL20 (green) and Hoechst (blue) in lesional skin from ACD patients (*n* = 4) and healthy individuals (*n* = 4). Scale bar, 50 μm. **(I)** Ear sections from K17 KO or WT mice challenged with OXA or ethanol as a control were stained with CCL20 (green) and Hoechst (blue). Scale bar, 20 μm. Data represent the mean ± SD. *p < 0.05, **p < 0.01, ***p < 0.001. All experiments were repeated for at least three times.

We then assessed whether K17 affects T cell recruitment by establishing a T cell-chemotaxis model *in vitro*. As shown by crystal violet staining, keratinocytes pretreated with mixed cytokines (IFN-γ, TNF-α, IL-17A, and IL-22) induced increased T cell trafficking; however, CD4^+^ and CD8^+^ T cell trafficking was significantly repressed upon siRNA silencing of *K17*, even after treatment with mixed cytokines (**Figure 3A**). Given the critical role of chemokines in T cell recruitment, we then screened chemokine levels by qRT-PCR in K17 KO and WT mice challenged with OXA *in vivo* (**Figure S3A**). Levels of CCL20, which is responsible for the chemotaxis of memory T cells, were markedly upregulated in K17 WT mice but decreased in K17 KO mice upon treatment with OXA (**Figure 3B**). Additionally, we verified these results in HaCaT cells treated with mixed cytokines and found that certain chemokines were significantly repressed in *K17* siRNA-transfected cells, with CCL20 representing the most significantly changed chemokine (**Figure 3C** and **Supplemental Figure S3B**). Thereafter, to further demonstrate the relationship between K17 and CCL20, we detected CCL20 expression in HaCaT cells transfected with pCMV6-XL5-K17 (LV K17) or *K17* siRNA to either overexpress or knockdown K17 *in vitro*, respectively. Following confirmation of the transfection efficiency by RT-PCR and western blot (**Supplemental Figures S3C, D**), we found that CCL20 levels were exclusively increased in K17-overexpressing cells but reduced in cells transfected with *K17* siRNA (**Figures 3D, E**), indicating that CCL20 expression was closely associated with K17, thus enhancing keratinocyte-mediated T cell chemotaxis. To further evaluate whether this chemotaxis was dependent on the chemokine CCL20, we neutralized the expression of CCL20 in K17-overexpressing HaCaT cells and detected CD4^+^ and CD8^+^ T cell trafficking *in vitro*. The results showed that K17-mediated CD4^+^ and CD8^+^ T cell chemotaxis was blocked upon treatment with the CCL20 mAb (**Figure 3F**), indicating that CCL20 plays an important role in keratinocyte-mediated T cell chemotaxis.

To obtain a deeper understanding of the contribution of CCL20 in the development of ACD, we analyzed the expression patterns of chemokines in the GEO datasets (GSE60028) and the microarray gene expression profile of skin biopsy samples from ACD patients. Consistent with our hypothesis, CCL20 levels were significantly higher in ACD patients compared with healthy control (**Figure 3G**). In parallel, we detected and compared the expression of CCL20 in clinical samples. Lesional skin epidermis of ACD patients and normal epidermis from healthy donors were detected and found that CCL20 was significantly increased in the lesional skin epidermis of ACD patients compared with normal skins (**Figure 3H**). Similar results were obtained from OXA-induced CHS mice. Specifically, CCL20 was markedly upregulated in K17 WT mice and scarcely undetectable in K17 KO mice challenged with OXA or K17 WT mice treated with EtOH (**Figure 3I**). These findings suggested that the substantial increase in CCL20 observed in ACD patients and OXA-induced mice was crucial for K17-mediated T cell infiltration.

### CCL20 Neutralization Alleviates OXA-Induced Skin Inflammation *In Vivo*

**Figure 4:**
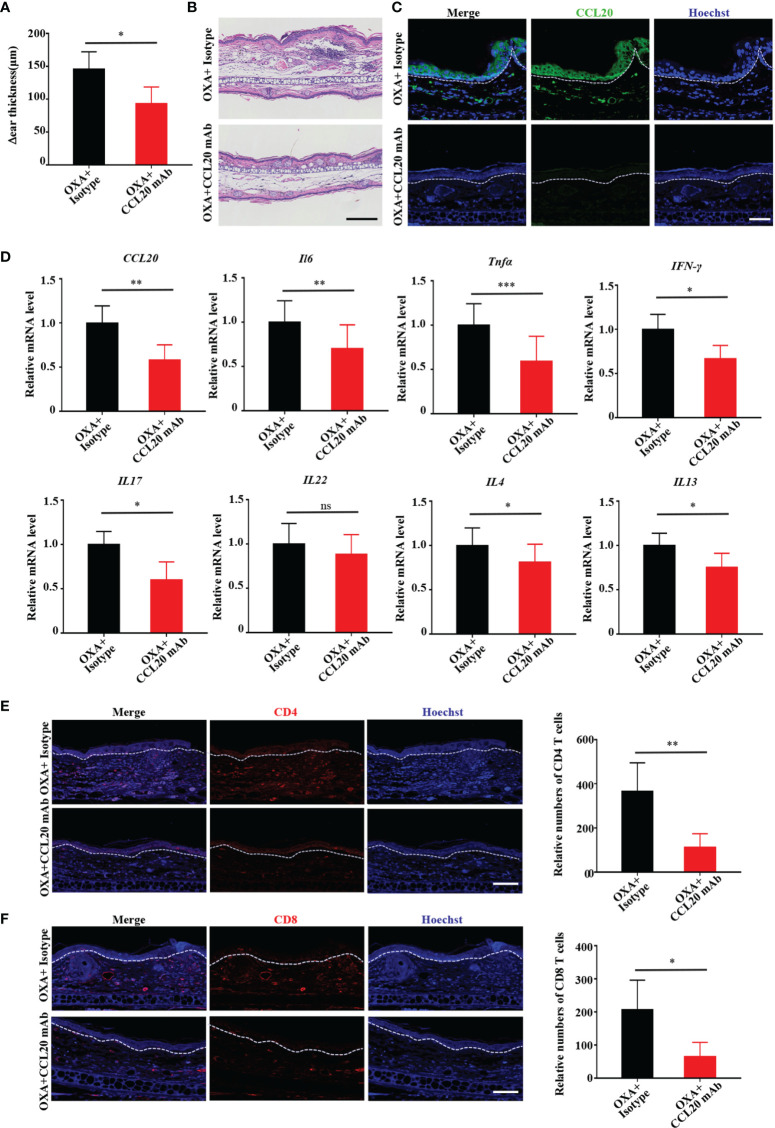
Neutralizing CCL20 alleviates OXA-induced ACD-like skin inflammation *in vivo.* **(A)** Examination of ear thickness in OXA-induced mice treated with or without the CCL20 mAb, *n* = 3 per group. **(B)** H&E staining of sections from OXA-induced mice treated with or without the CCL20 mAb. Scale bar, 100 μm, *n* = 3 per group. **(C)** Immunofluorescen-ce staining for CCL20 (green) and Hoechst (blue) from OXA-induced mice treated with or without the CCL20 mAb. Scale bar, 20 μm, *n* = 3 per group. **(D)** Relative *CCL20, Il6*, *Tnfa*, *IFN-γ*, *Il17*, *Il22*, *Il4* and *Il13* mRNA expression in epidermal tissues from mouse biopsies was analyzed by qRT-PCR, *n* = 3 (mean ± SD). **(E, F)** Immunofluorescence staining for CD4 and CD8^+^ T cells and relative numbers in sections from OXA-induced mice treated with or without the CCL20 mAb. CD4 (red), CD8 (red), and Hoechst (blue). Scale bar, 20 μm. Data represent the mean ± SD (*n* = 3). *p < 0.05, **p < 0.01, ***p < 0.001, ns, not significant. All the bars represent at least average of three independent experiments.

To further ascertain whether CCL20 is necessary for ACD development *in vivo*, we neutralized CCL20 with CCL20 mAb prior to sensitization with OXA on one ear, with the opposite ear receiving isotype application. Ear thickness was measured before injection and after OXA challenge 5 days later. Consistent with the results obtained from the K17 KO mice, inhibiting CCL20 dramatically reduced epidermal thickness in response to OXA, whereas isotype-injected ears failed to exhibit any significant change (**Figure 4A**). Additionally, H&E staining revealed weaker inflammation and less ear swelling than those in the control (**Figure 4B**). Moreover, immunofluorescence and RT-PCR results showed a significant reduction in both the protein and mRNA levels of CCl20 in mice treated with the CCL20-neutralizing antibody (**Figures 4C, D**). Concurrently, the mRNA levels of proinflammatory cytokines, including *Il6*, *Tnfa*, *IFN-γ*, *Il17*, *Il22*, *Il4* and *Il13*, chemokines *CCL17, CCL27, CXCL1, CXCL9, CXCL10, CXCL11* (**Figures 4D** and **Supplemental Figure S4A**) were repressed upon neutralization of CCL20. Furthermore, mice administered with the CCL20 mAb were also displayed a reduction in CD4^+^, CD8^+^, and CD3^+^ T cell infiltration (**Figures 4E, F** and **Supplemental Figure S4B**), indicating that CCL20 is crucial for the development of the OXA-induced CHS response *in vivo*.

### K17-Mediated Upregulation of CCL20 Expression Depends on Its Nuclear Translocation

**Figure 5:**
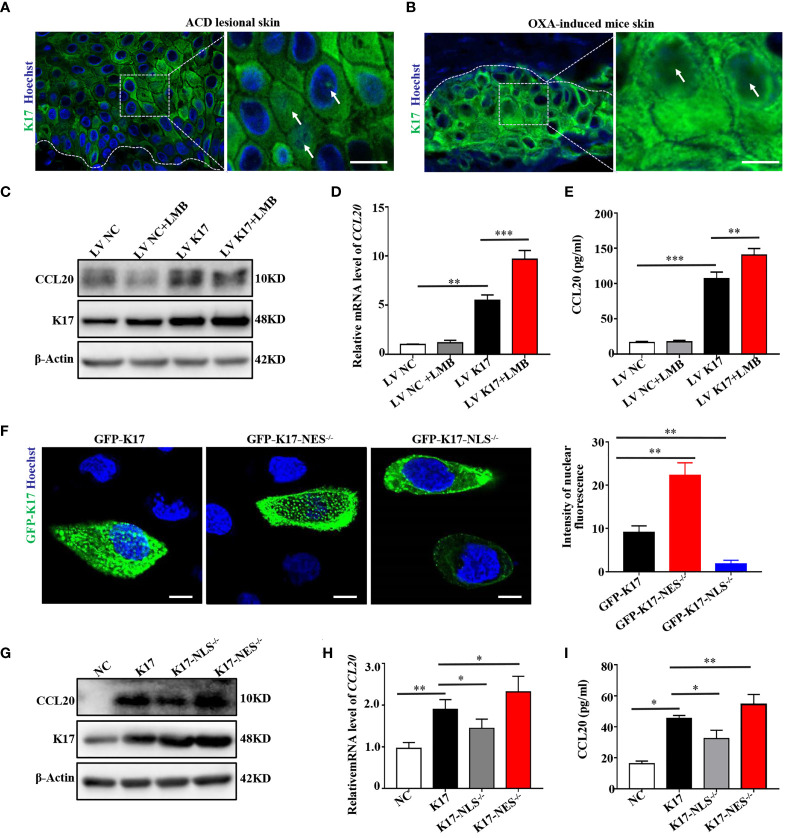
K17 upregulates CCL20 expression dependent on its nuclear translocation. Immunofluorescence staining for K17 located in the nucleus of keratinocytes in lesional skin from **(A)** ACD patients (*n* = 3) and **(B)** OXA-induced mice (*n* = 3). K17 (green) and Hoechst (blue). Scale bar, 10 μm. **(C)** K17 and CCL20 protein levels were determined using western blot. **(D)** Relative *CCL20* mRNA expression analyzed by qRT-PCR. **(E)** CCL20 levels secreted into HaCaT cell culture medium were measured by ELISA. **(F)** Immunofluorescence staining of K17 distributed in the nuclei of HaCaT cells transfected with WT GFP-K17 and mutant GFP-K17 and the intensity of nuclear fluorescence per unit area. Scale bar, 10 μm. **(G)** K17 and CCL20 levels were analyzed by western blot. **(H)** Relative *CCL20* mRNA expression was analyzed by qRT-PCR. **(I)** CCL20 levels secreted into HaCaT cell culture medium were measured by ELISA. Data represent the mean ± SD (*n* = 3). *p < 0.05, **p < 0.01, ***p < 0.001. All experiments were repeated for at least three times.

Recent emerging evidence has revealed that K17 is not exclusively present in the cytoplasm but also localizes to the nucleus (20). Consistent with these findings, K17 was overexpressed, and a subset of the overall K17 population localized to the nucleus of epidermal keratinocytes in the lesional skin of ACD patients and OXA-challenged mice (**Figures 5A, B**). To investigate whether CCL20 expression was associated with the translocation of K17, we detected CCL20 expression in K17-overexpressing HaCaT cells treated with or without LMB, a nuclear transport inhibitor, to block the nuclear export of K17. We confirmed that LMB treatment significantly upregulated the nuclear accumulation of K17 (**Supplemental Figure S5A**). Additionally, western blot, qRT-PCR, ELISA, and immunofluorescence results showed a marked increase in CCL20 expression in K17-overexpressing HaCaT cells, and CCL20 expression was further upregulated upon blockade of K17 nuclear transport with LMB *in vitro* (**Figures 5C–E**).

Hobbs et al. (21) reported that K17 harbors a classical bipartite NLS and an evolutionarily conserved NES sequence based on truncation mutant analysis. Accordingly, we constructed a K17-overexpressing vector (pEGFP-N1-K17) fused with green fluorescent protein and two mutant expression vectors lacking the NLS (aa 385–400) or NES (aa 394–399) sequence, transfected these vectors into HaCaT cells, and investigated the subcellular localization of the encoded K17 proteins by confocal fluorescence microscopy and time-lapse live-cell imaging. Notably, a portion of the GFP-K17 fusion protein exhibited diffuse or punctate patterns in the nucleus following transfection of HaCaT cells with the K17-GFP-overexpressing vector for 24 h. Moreover, nuclear immunofluorescence intensity of the diffuse or punctate pattern of intranuclear K17 was dramatically upregulated in the absence of the NES sequence but minimally present in the nucleus in the absence of the NLS sequence (**Figure 5F** and **Supplemental Figure S5B**). These results demonstrated that the translocation of K17 was dependent on the NLS and NES sequences. We then performed western blot, qRT-PCR, and ELISA to detect CCL20 levels in HaCaT cells transfected with the K17-overexpressing vector or one of the mutant K17 vectors. We found that CCL20 expression was further elevated when the NES sequence was deleted, whereas decreased in the presence of K17 lacking the NLS sequence (**Figures 5G–I**), which is consistent with the results from treatment with LMB. These results indicated that elevated K17 expression and its subcellular localization are essential for the upregulation of CCL20, and this process is dependent on the NLS and NES sequences.

### Nuclear Translocation of K17 Enhances the Interaction With STAT3 and Promotes Its Activation to Upregulate CCL20 Expression

**Figure 6:**
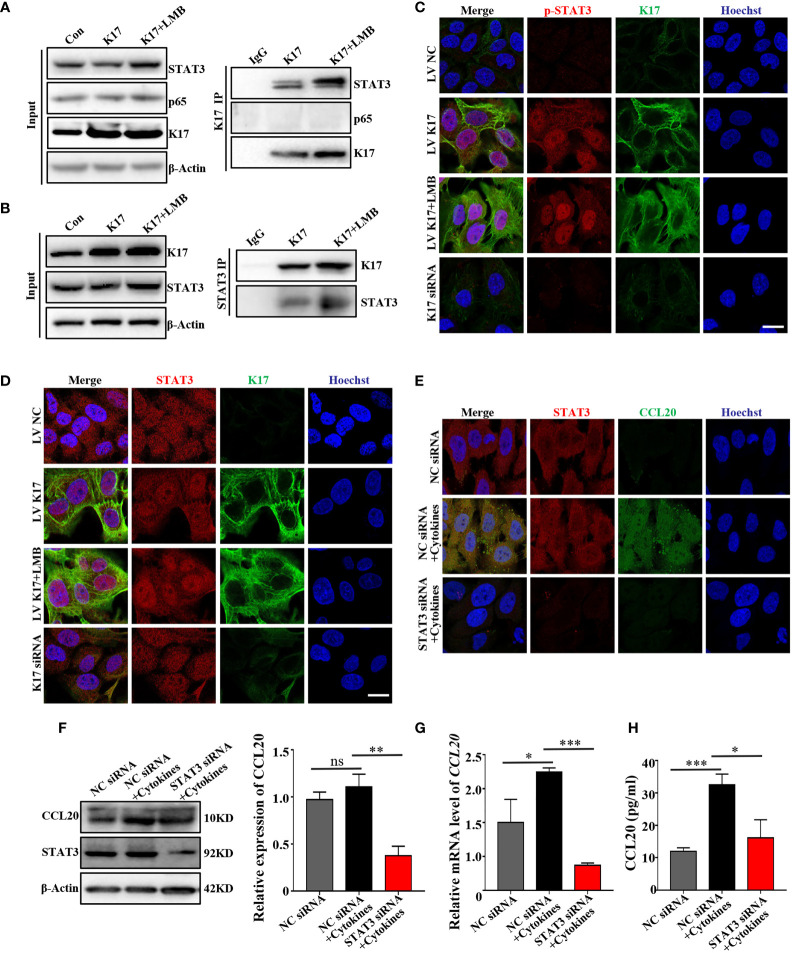
Nuclear translocation of K17 enhances the interaction with STAT3 and promotes STAT3 activation to upregulate CCL20 expression. Cells were co-immunoprecipitated with **(A)** anti-K17 and **(B)** anti-STAT3 and detected by western blot. **(C)** Immunofluorescence staining of STAT3 (red), K17 (green) and Hoechst (blue). Scale bar, 10 μm. **(D)** Immunofluorescence staining of p-STAT3 (red), K17 (green) and Hoechst (blue). Scale bar, 10 μm. **(E)** Immunofluorescence staining of CCL20 (green), STAT3 (red) and Hoechst (blue). Scale bar, 10 μm. **(F)** CCL20 level was analyzed by western blot. **(G)** Relative *CCL20* mRNA expression was analyzed by qRT-PCR. Data represent the mean ± SD (*n* = 3). **(H)** CCL20 levels secreted into keratinocytes cell culture medium were measured by ELISA. Data represent the mean ± SD (*n* = 3). *p < 0.05, **p < 0.01, ***p < 0.001, ns, not significant. All experiments were repeated for at least three times.

Given that CCL20 transcription could be promoted by activation of STAT3 and nuclear factor-kappaB (23, 24). We then investigated whether these proteins participate in the regulation of CCL20 mediated by K17. Co-IP experiments using anti-K17 revealed that K17 interacted with STAT3 rather than p65 (**Figure 6A**). Moreover, LMB-mediated blockade of K17 nuclear transport resulted in elevated levels of K17 interaction with STAT3. Similar results were also found in the Co-IP experiment with anti-STAT3 (**Figure 6B**), indicating that K17 interacts with STAT3 rather than p65 to regulate CCL20 expression. Thereafter, to gain a further insight into the molecular mechanism between K17 and STAT3, we evaluated the activation and nuclear translocation of STAT3 upon blockade of K17 nuclear transport with LMB. Immunolocalization results showed that K17 colocalized with STAT3 in the cytoplasm and around the nucleus, both LMB-mediated blockade of K17 nuclear transport group and K17-NES^-/-^ group showed significantly upregulated the nuclear accumulation of STAT3 (**Figure 6C** and **Supplemental Figure S6A**). Moreover, phosphorylation of STAT3 was also further elevated in LMB-treated K17-overexpressing cells (**Figure 6D**). Given that STAT3 could combine with K17 and regulate the transcription of CCL20, we then evaluated CCL20 expression in keratinocytes transfected with STAT3 siRNA, and evaluated the interference efficiency by western blot and qRT-PCR (**Supplemental Figure S6B**). Western blot, immunofluorescence staining, qRT-PCR, and ELISA revealed that CCL20 levels were markedly repressed in keratinocytes transfected with STAT3 siRNA compared with those transfected with NC siRNA upon simultaneously stimulation with mixed cytokines (**Figures 6E–H**). Together, our data revealed the novel role of K17, which could directly interact with STAT3 in the cytoplasm and around the nucleus, facilitating the nuclear translocation and activation of STAT3 to promote the transcription of CCL20.

## Discussion

In the present study, we evaluated the crucial role of K17 in the pathogenesis of ACD and explored the underlying mechanism both *in vivo* and *in vitro*. Our data demonstrated that K17 may play a critical role in priming keratinocyte-mediated T cell trafficking and aggravate the CHS response through induction of CCL20 expression. Furthermore, we found that K17 could translocate into the nucleus through a process dependent on the NLS and NES sequences. Nuclear translocation of K17 further potentiated the interaction between K17 and STAT3, which subsequently activated the STAT3 signaling pathway and induced excessive production of CCL20, resulting in increased T cell infiltration and severe skin inflammation.

K17 serves as a key regulator and is broadly considered to be involved in several types of T cell-mediated human inflammatory skin diseases. Additionally, the K17 level is regulated by various inflammatory cytokines, including IL-17, IL-22, TNF-α, and IFN-γ (25, 26). However, whether K17 participates in the immunological mechanisms of ACD remains poorly understood. Here, we observed increased K17 expression in both ACD patients and an experimental model of OXA-induced mice and that mice lacking K17 exhibited a weaker skin inflammatory response as compared with WT controls. These data demonstrated a pivotal role of K17 in ACD development; however, a deeper understanding of the immune cells and signaling pathways involved in this response is needed. Furthermore, whether K17 inhibition represents a therapeutic approach for ACD and other inflammatory/autoimmune diseases should be further assessed.

ACD patients often suffer from severe and chronic pruritus, with pruritus-induced mechanical scratching capable of exacerbating atopic skin inflammation (27, 28). Recent studies have revealed that mechanical scratching stimulates epidermal keratinocytes release of the chemokine CCL20, which selectively binds to its receptor C–C motif chemokine receptor 6 and attracts effector/memory T cells (29). In the present study, CCL20 expression was increased in epidermal keratinocytes of lesional skin from ACD patients and OXA-induced mice. Moreover, inhibiting CCL20 significantly suppressed the infiltration of CD4^+^ and CD8^+^ T cells. These observations strongly illustrate the critical role of CCL20 in the development of ACD-like skin inflammation. Furthermore, according to results from experiments involving the CCL20 mAb, CCL20-neutralizing antibodies that suppress skin inflammation might exhibit efficacy in the future as a treatment for ACD. In addition to CCL20, the expression of multiple chemokines (CCL17, CCL27, CXCL9, CXCL10, CXCL11) and chemokine receptors (CCR4, CXCR3) were also repressed in K17 KO mice upon treatment with OXA and K17-siRNA-transfected HaCaT cells, suggesting that other chemokines related to K17 may also participate in the development of ACD through specific signaling pathways like as CCL20.

Keratins are not simply intracellular skeletal structures but rather exhibit functional roles in modulating the immune response (30, 31). Specifically, in recent years, type I K17 was identified as present in the nucleus and involved in altering the nuclear localization and function of 14-3-3δ, the cell cycle inhibitor p27^KIP1^, and the heterogeneous ribonucleoprotein hnRNP K, suggesting a possible role for K17 in protein synthesis, proliferation of carcinoma cells, and inflammatory gene expression (32–34). Although few studies have focused on the nuclear translocation of K17, studies investigating cervical and skin tumors hint at the promising prospect that nuclear K17 might perform multiple roles in multiple diseases, which is similar to its function in the cytoplasm. Interestingly, in the present study, we observed that K17 translocated to the nucleus of keratinocytes from ACD patients and OXA-induced mice. Moreover, we verified that K17 translocation was regulated by NLS and NES sequences *via* time-lapse live-cell imaging, which provided an accurate quantification of single-cell dynamics. These results further confirmed the notion of nuclear-localized cytoskeletal proteins, including the actin-binding protein α-actin, and select keratins, such as K7, K8, and K18, which dynamically shuttle between the cytoplasm and nucleus in cultured HeLa cells or murine fibroblasts (20). Additionally, we found that nuclear K17 exhibited a punctate and diffuse pattern in keratinocytes from ACD patients and HaCaT cells transfected with pEGFP-N1-K17. This phenomenon was previously reported in A431, HeLa, and BT-20 cells (35, 36). Nuclear K17 exhibits a distinct form compared with that observed in the cytoplasm, with these differences possibly regulated by posttranslational modifications (e.g., phosphorylation, acetylation, or succinylation) or interactions with select proteins, this finding requires further investigation in our future studies.

Increased K17 expression in psoriatic lesional skin upregulates the expression of multiple proinflammatory cytokines and chemokines, including IFN-γ, IL-22, and CXCL1, and plays an important role in the development of psoriasis (37, 38). In the present study, CCL20 expression was closely related to K17 expression and further enhanced when K17 nuclear export was blocked with the nuclear transport inhibitor LMB or by deleting the NES sequence. These results strongly support our hypothesis that K17 translocates into the nucleus and upregulates CCL20 expression, thus aggravating skin inflammation. Based on these results, K17 potentially represents a target for treating ACD. Additionally, Chung et al. (39) observed an interaction between K17 and the heterogeneous ribonucleoprotein hnRNP K that plays a particularly prominent role in regulating the expression of the inflammatory chemokines CXCL9, CXCL10, and CXCL11 as a part of the growth-promoting signature in various types of cancers. Additionally, our group uncovered that K17 was a direct interactor of STAT3, and K17 ubiquitination could promote STAT3 activation in pso-mix-treated HaCaT cells (40). Consistently, we observed that K17 combined with STAT3 in the cytoplasm and around the nucleus, accelerating the translocation and activation of STAT3. Meanwhile, both blockade of K17 nuclear export with LMB and deleted the nuclear export sequence of K17 are showed enhanced nuclear accumulation of STAT3 and upregulated expression of CCL20, indicating that K17 interact with STAT3 in the cytoplasm around the nucleus, facilitating the activation and phosphorylation of STAT3, thus inducing the upregulation of CCL20. Translocation of K17 into nucleus may play multifunctional roles in numerous biological processes and occupies a central position in many disease mechanisms like as cytoplasm K17. Moreover, nuclear translocation of K17 may be associated with its ubiquitination or other posttranslational modifications, which needs to be explored in our future study.

In conclusion, increased K17 expression induced the production of CCL20, which was further increased by nuclear translocation of K17 through a process dependent on the NLS and NES sequences. These events increased the infiltration of CD4^+^ and CD8^+^ T cells, thereby amplifying the inflammatory response and promoting ACD development. These findings significantly extend our previous understanding of the underlying pathogenesis of ACD and provide a potential therapeutic approach for ACD and other allergen-induced inflammation related skin diseases.

## Data Availability Statement

The raw data supporting the conclusions of this article will be made available by the authors, without undue reservation.

## Ethics Statement

The studies involving human participants were reviewed and approved by Fourth Military Medical University. The patients/participants provided their written informed consent to participate in this study. The animal study was reviewed and approved by Fourth Military Medical University. Written informed consent was obtained from the owners for the participation of their animals in this study.

## Author Contributions

YL, ZZ, BL, XB, HF, ED, and GW conceptualized the study. YL wrote the manuscript. JC and CZ recruited the patients. YL, ZZ, BL, XB, HF, DZ, JC, ED, and GW contributed to the generation and/or analyses of the data. All authors read the manuscript and contributed to the discussions and revision. All authors contributed to the article and approved the submitted version.

## Funding

This work was supported by grants from the National Natural Science Foundation of China (nos. 82003341, 81872519, 82073435) and Shaanxi scientific research grant (2020JM-320).

## Conflict of Interest

The authors declare that the research was conducted in the absence of any commercial or financial relationships that could be construed as a potential conflict of interest.

## Publisher’s Note

All claims expressed in this article are solely those of the authors and do not necessarily represent those of their affiliated organizations, or those of the publisher, the editors and the reviewers. Any product that may be evaluated in this article, or claim that may be made by its manufacturer, is not guaranteed or endorsed by the publisher.
