# Supplementary material for: Keratin 17 Promotes T Cell Response in Allergic Contact Dermatitis by Upregulating C–C Motif Chemokine Ligand 20
Source: Front Immunol. 2022 Feb 1;13:764793. doi: 10.3389/fimmu.2022.764793 (PMC8845002; doi:10.3389/fimmu.2022.764793)
Supplement: Supplementary file 1 [file DataSheet_1.pdf]

Supplementary Material

Supplementary Tables

Table S1. siRNA sequences used in this study.

**Gene symbol**		**siRNA Sequences**
Human K17 siRNA	Sense	CCTGACTCAGTACAAGAAATT
	Anti-sense	UUUCUUGUACUGAGUCAGGTT
Human STAT3 siRNA	Sense	AGUCAGGUUGCUGGUCAAATT
	Anti-sense	UUUGACCAGCAACCUGACUTT
NC siRNA	Sense	UUCUCCGAACGUGUCACGUTT
	Anti-sense	ACGUGACACGUUCGGAGAATT

Table S2. Primers for the analysis of mRNAs

Primer name		sequence 5′→3′
Human K17	Forward	CCAGCTCAGCATGAAAGCATC
	Reverse	ACCTCTTCCACAATGGTACGC
Human CCL20	Forward	TCCTGGCTGCTTTGATGTCA
	Reverse	CAAAGTTGCTTGCTGCTTCTGA
Human CXCL9	Forward	GAGTGCAAGGAACCCCAGTAGT
	Reverse	TTGTAGGTGGATAGTCCCTTGGTT
Human CXCL10	Forward	TTCAAGGAGTACCTCTCTCTAG
	Reverse	CTGGATTCAGACATCTCTTCTC
Human CXCL11	Forward	GACGCTGTCTTTGCATAGGC
	Reverse	GGATTTAGGCATCGTTGTCCTTT
Human CXCL1	Forward	AGGGAATTCACCCCAAGAAC
	Reverse	ACTATGGGGGATGCAGGATT
Human CCR6	Forward	ATGCGGTCAACTTTAACTGTGG
	Reverse	CCCGGAAAGATTTGGTTGCCT
Human CCR4	Forward	CCTTGCCATCTCGGATCTGC
	Reverse	AGACCTAGCCCAAAAACCCAC
Human CXCR3	Forward	GGTCATGGCCTACTGCTATGC
	Reverse	CCACGTCTACCCTGCTTTCT
Human CCL17	Forward	TACCATGAGGTCACTTCAGATGC
	Reverse	GCACTCTCGGCCTACATTGG
Human CCL27	Forward	GACAGAGATACCGCCACGTT
	Reverse	CAGTGTTCTTGCCTTGGCTC
Human β-Actin	Forward	GGCTACAGCTTCACCACCAC
	Reverse	TGCGCTCAGGAGGAGC
Mouse K17	Forward	GCCCACCTGACTCAGTACAA
	Reverse	GGAGCTGAGTCCTTAACGGG
Mouse CCL20	Forward	CAGGCAGAAGCAAGCAACTAC

	Reverse	AGCTTCATCGGCCATCTGTC
Mouse CXCL9	Forward	TGGCTGGGATTCACCTCAAG
	Reverse	CCGTTACTTGGGGACACCTT
Mouse CXCL10	Forward	GACAGAGATACCGCCACGTT
	Reverse	CAGTGTTCTTGCCTTGGCTC
Mouse CXCL11	Forward	GCCCTACGGTGGAAGTCATA
	Reverse	GAACACTGGCCGTTCTTTCC
Mouse TNFα	Forward	CCCTCACACTCACAAACCAC
	Reverse	ACAAGGTACAACCCATCGGC
Mouse IL6	Forward	CGGAGAGGAGACTTCACAGAG
	Reverse	ATTTCCACGATTTCCCAGAG
Mouse IFN-γ	Forward	ACTCATACACCAGGTCACGC
	Reverse	CAGTGTAAAGGTGCACATGACG
Mouse IL17	Forward	ACTCCTGGGAAGACCTCATTGG
	Reverse	GGCCACATGGTGGACAAT CG
Mouse IL22	Forward	CCCTATATCACCAACCGCAC
	Reverse	GCTCACTCATACTGACTCCGT
Mouse IL4	Forward	TTGAACAGCCTCACAGAGCAGA
	Reverse	GTTGTGTTCTTGGAGGCAGCA
Mouse IL13	Forward	CTAACCTCATTCCCCAACCA
	Reverse	CTCAGCCTCCCAAAGTGCT
Mouse CCR6	Forward	CCTGGGCAACATTATGGTGGT
	Reverse	CAGAACGGTAGGGTGAGGACA
Mouse CCR4	Forward	GGAAGGTATCAAGGCATTTGGG
	Reverse	GTACACGTCCGTCATGGACTT
Mouse CXCR3	Forward	TACCTTGAGGTTAGTGAACGTCA
	Reverse	CGCTCTCGTTTTCCCCATAATC
Mouse CCL17	Forward	GACGACAGAAGGGTACGGC
	Reverse	GCATCTGAAGTGACCTCATGGTA

Mouse CCL27	Forward	GCAGCATTCCTACTGCCAC
	Reverse	AGGTGAAGCACGAAAGCCTG
Mouse CXCL1	Forward	TGGCTGGGATTCACCTCAAG
	Reverse	CCGTTACTTGGGGACACCTT
Mouse β-Actin	Forward	TGGATACAGGCCAGACTTTG
	Reverse	GATTCAACTTGCGCTCATCTTA

Table S3. Complete list of the detailed clinical information about ACD patients in this study

Patient #	Gender	Age	Confirm method	Skin biopsy time
1800974	F	41	Positive-patch tests	4 days
1800975	M	38	Positive-patch tests	10 days
1510796	F	52	Positive-patch tests	4 days
2106337	F	38	Positive-patch tests	1 mouth
1911409	M	33	Positive-patch tests	7 days
1804831	M	68	Positive-patch tests	1mouth

Table S4. Complete list of the detailed clinical information about ACD patients from GEO dataset

Patient #	Gender	Age	Allergen group[a]	Reaction[b]
1	F	32	Nickel	+2
2	F	45	Rubber	+1
3	M	31	Fragrance	+2
4	F	33	Nickel	+1
5	M	42	Nickel	+2
6	M	52	Nickel	+2
7	F	48	Other Metals	+3
8	F	37	Nickel	+1
9	M	54	Rubber	+2
10	F	33	Nickel	+1
11	F	39	Rubber	+2
12	M	57	Other Metals	+1
13	M	54	Fragrance	+1

a: Allergens were grouped into four categories: 10 nickel, fragrance (2 Balsam of Peru, 1 Fragrance Mix), rubber (5 Carba mix, 2 Thiuram), and other metals (3 Cobalt, 1 Potassium Dichromate)

b: Positive-patch tests were graded according to established guidelines

Supplementary Figures

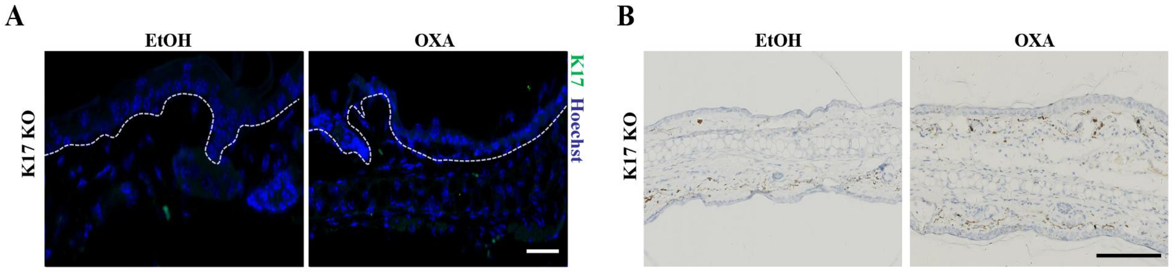

Supplementary Figure 1. K17 levels in the lesional skin of K17 KO mice. (A) Immunofluorescence staining for K17 (green) and Hoechst (blue) in ear sections from K17 KO mice challenged with OXA or ethanol as a control. Scale bar, 10 μm, *n* = 3 per group. **(B)** Representative images of immunohistochemical staining for K17 in ear skin from K17 KO mice induced with OXA, with ethanol used as a control. Scale bar, 100 μm, *n* = 3 per group. All experiments were repeated for at least three times.

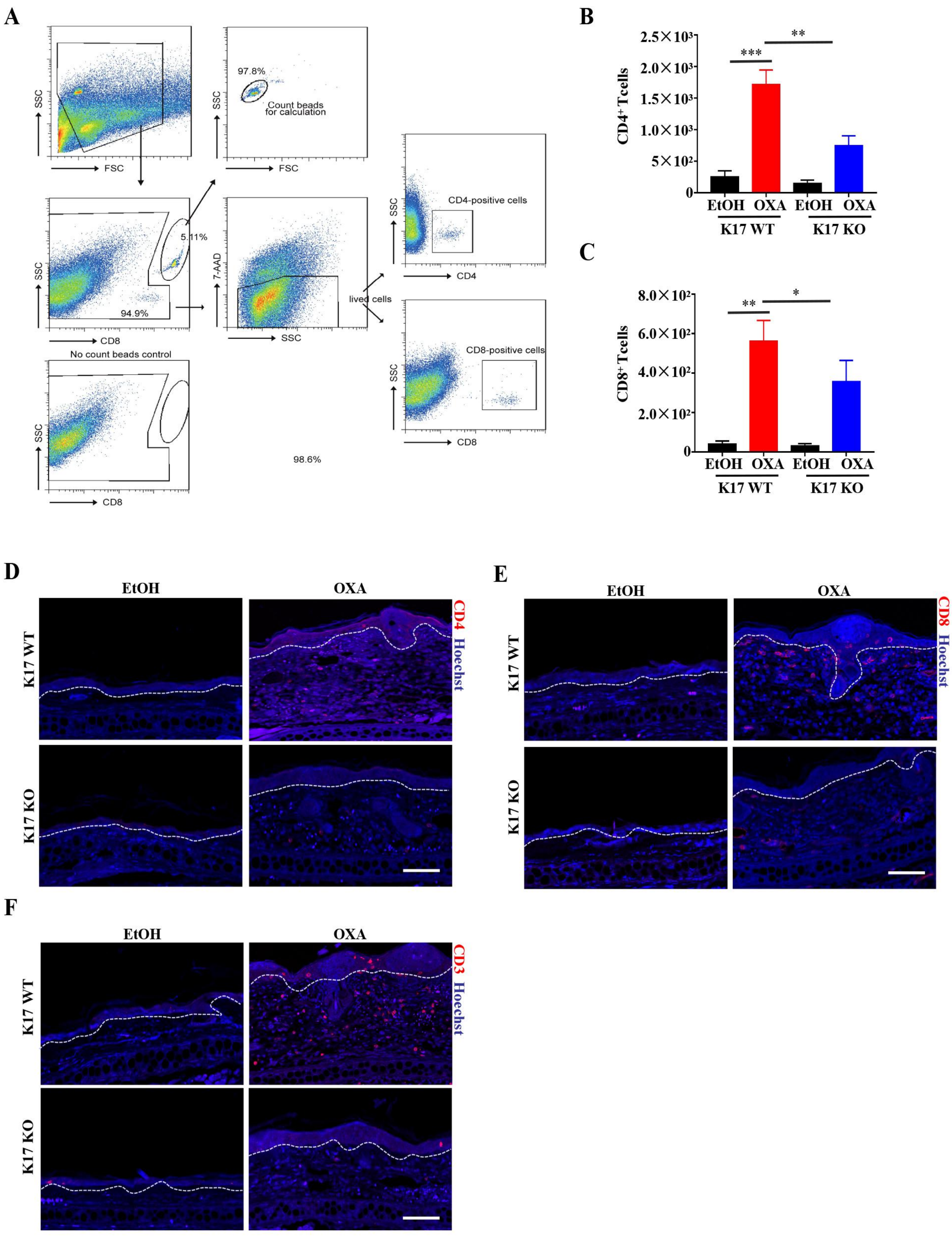

Supplementary Figure 2. Gating strategy for flow cytometry and T cell infiltration in ear tissue from K17 WT and K17 KO mice. (A) Single-cell suspensions were prepared from the ears of mice, digested with collagenase type IV and DNase I, and stained with anti-CD4 and anti-CD8 antibodies. CountBright absolute counting beads were used to calculate the absolute

cell numbers. Dead cells were identified by 7-AAD staining. Cell events were gated by using forward scatter (FSC) and side scatter (SSC) plots to exclude debris. Counting beads were identified based on plots of the fluorescence intensity, and cell densities and numbers were calculated. 7-AAD-negative live cells were subjected to analysis of CD4 and CD8 expression. Absolute numbers of **(B)** $CD4^+$ T cells and **(C)** $CD8^+$ T cells were calculated and analyzed based on flow cytometric analyses of (Figure 2E-F). **(D-F)** Ear sections from K17 KO or WT mice challenged with OXA were stained with CD4 (red), CD8 (red), CD3 (red), and Hoechst (blue). Ethanol was used as a control. Scale bar, 20 µm, $n = 3$ per group. Data represent the mean ±SD ($n = 3$). $*p < 0.05$, $**p < 0.01$, $***p < 0.001$. All experiments were repeated for at least three times.

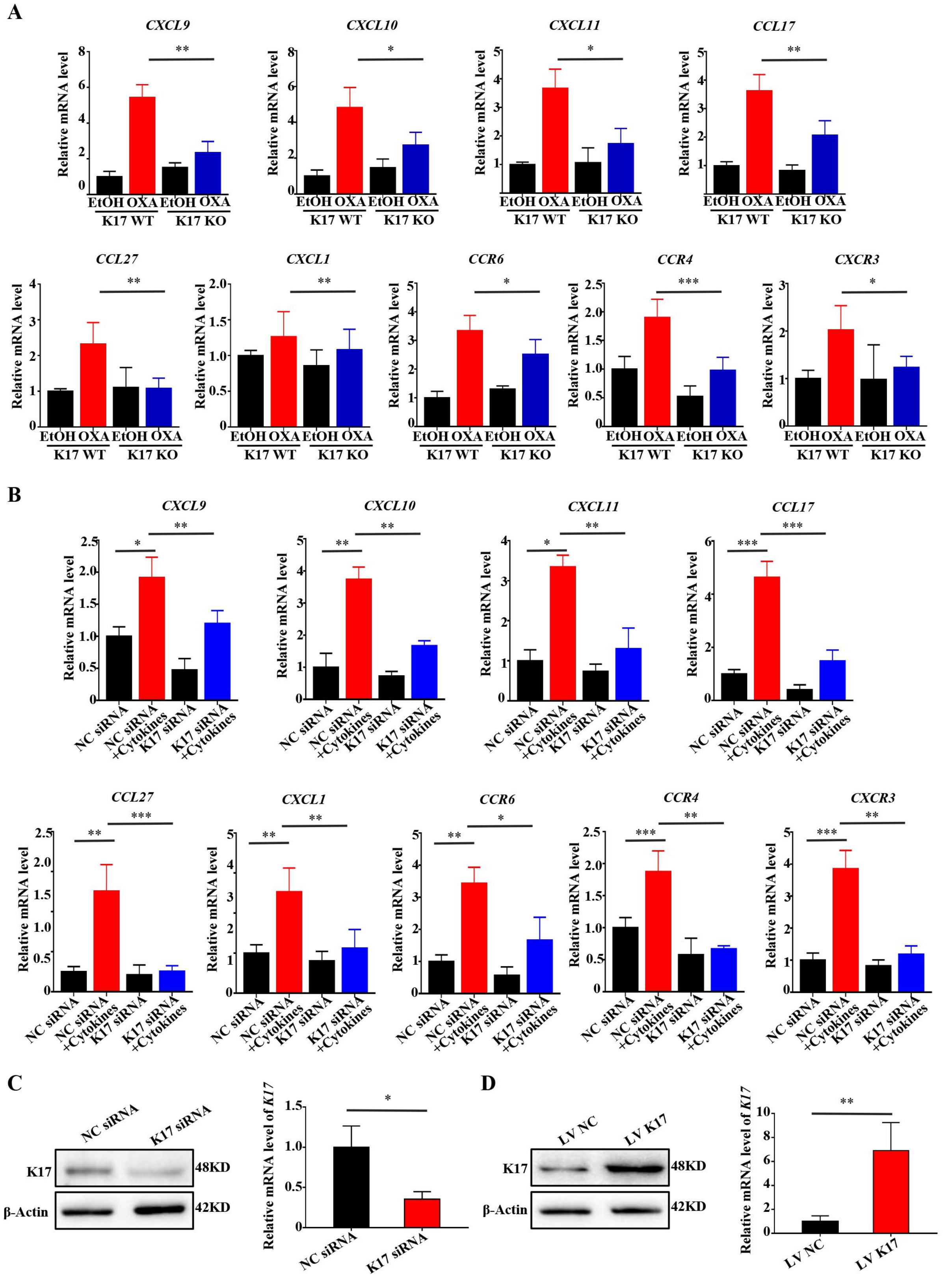

Supplementary Figure 3. Chemokine screening and K17 overexpression or knockdown efficiency. (A) Relative mRNA expression of chemokines and chemokine receptors in K17 KO and WT mice treated with OXA was analyzed by qRT-PCR, *n* = 3 per group. **(B)** Relative

mRNA expression of chemokines and chemokine receptors in cytokine-treated or *K17*-siRNA-transfected HaCaT cells was analyzed by qRT-PCR. Data are expressed as means ± SD (n = 3). **(C)** The knockdown efficiency of *K17* siRNA was analyzed using western blot and qRT-PCR. Data are expressed as means ± SD (n = 3). **(D)** The transfection efficiency of pCMV6-XL5-K17 was analyzed using western blot and qRT-PCR. Data represent the mean ± SD (n = 3). *$p < 0.05$, **$p < 0.01$, ***$p < 0.001$. All experiments were repeated for at least three times.

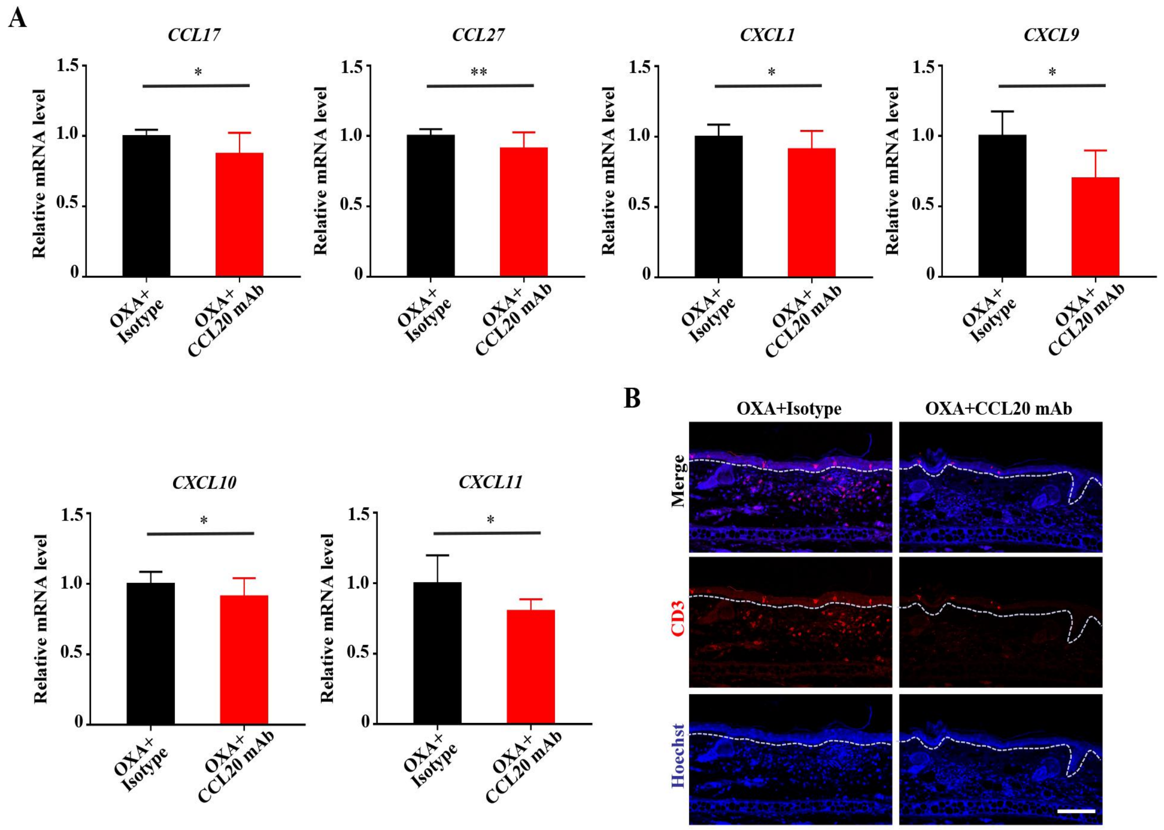

Supplementary Figure 4. CCL20 blockade reduces the expression of chemokines and the infiltration of CD3⁺ T cells. (A) Relative mRNA expression of chemokines in OXA-induced mice treated with or without the CCL20 mAb, *n* = 3 per group. **(B)** Immunofluorescence staining for CD3 (red) and Hoechst (blue) in OXA-induced mice treated with or without the CCL20 mAb. Scale bar, 20 μm. *n* = 3 per group, $*p < 0.05$, $**p < 0.01$. All experiments were repeated for at least three times.

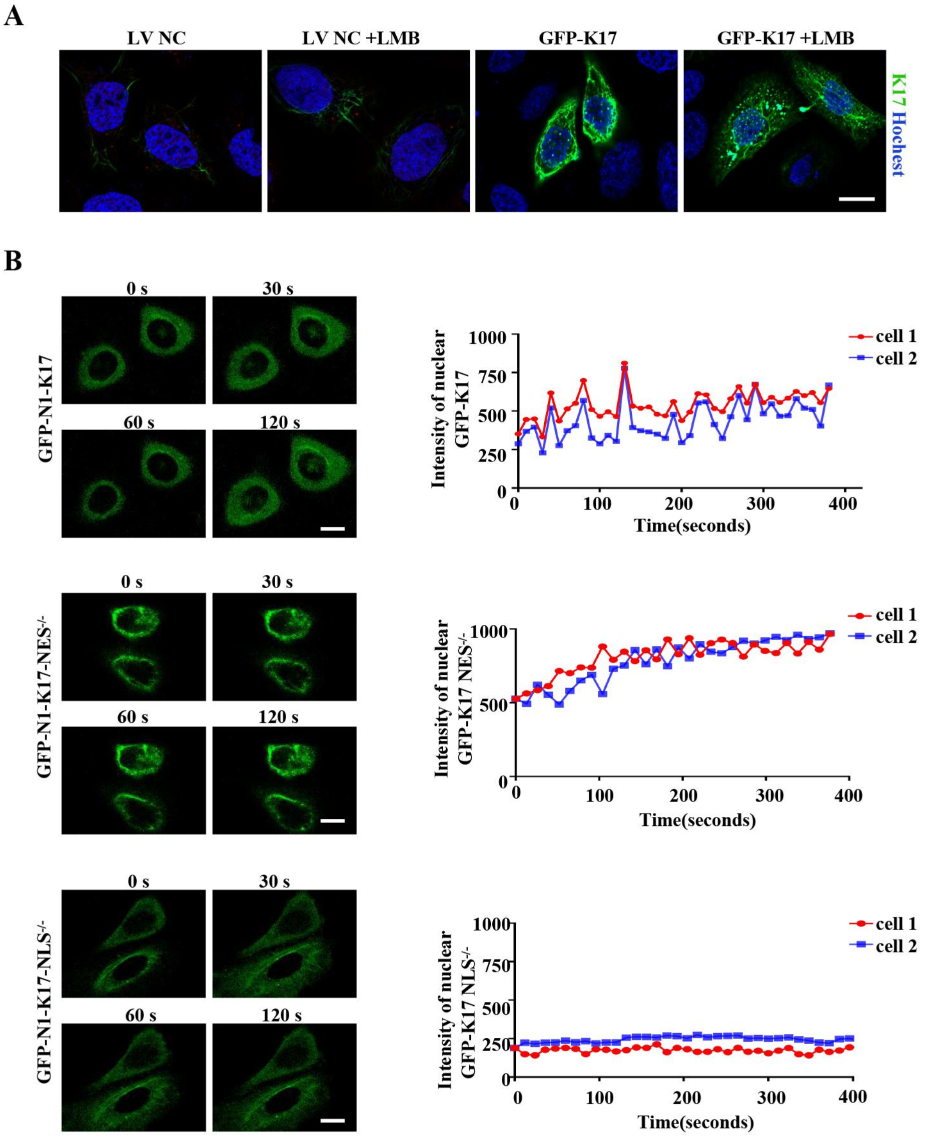

Supplementary Figure 5. Nuclear localization and nuclear import and export sequences of K17. (A) Immunofluorescence staining for nuclear GFP-K17 in cells treated with or without LMB. Scale bar, 10 µm. **(B)** Time-lapse living cell imaging analysis for GFP-K17, GFP-K17-NES$^{-/-}$, GFP-K17-NLS$^{-/-}$ and single-cell traces of nuclear K17 generated using Image-Pro Plus software. Data represent the mean ± SD (*n* = 3). All experiments were repeated for at least three times.

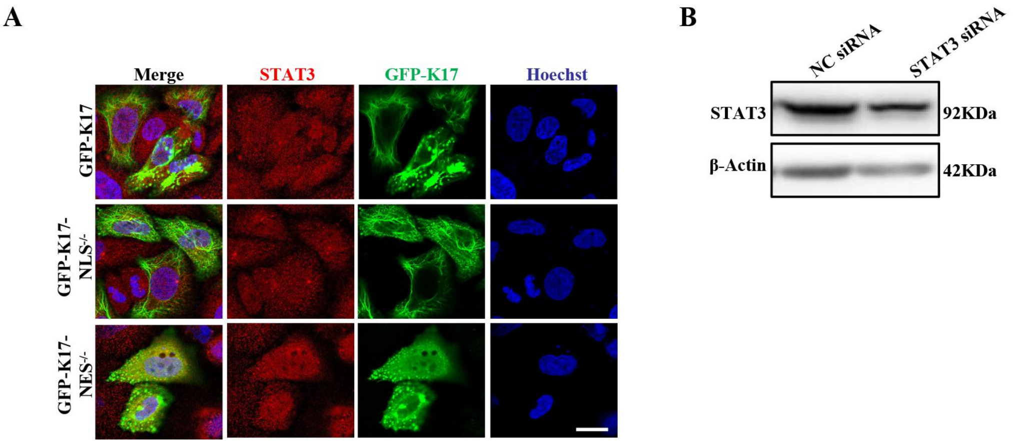

Supplementary Figure 6. Tanslocation of K17 facilitate the nuclear accumulation of STAT3 and the knockdown efficiency of STAT3 siRNA. (A) Immunofluorescence staining in WT GFP-K17 and mutant GFP-K17 transfected keratinocytes. STAT3 (red), K17 (green) and Hoechst (blue). Scale bar, 10 μm. **(B)** The knockdown efficiency of STAT3 siRNA was analyzed by western blot. All experiments were repeated for at least three times.
